# Kinetics and Mechanism of In Situ Metallization of Bulk DNA Films

**DOI:** 10.1186/s11671-022-03658-8

**Published:** 2022-01-24

**Authors:** Zi-Hao Shi, Feng-Ming Hsu, Bradley W. Mansel, Hsin-Lung Chen, Ljiljana Fruk, Wei-Tsung Chuang, Yu-Chueh Hung

**Affiliations:** 1 Institute of Photonics Technologies, National Tsing Hua University, Hsin Chu, Taiwan; 2 Department of Chemical Engineering, National Tsing Hua University, Hsin Chu, Taiwan; 3 Department of Chemical Engineering and Biotechnology, University of Cambridge, London, UK; 4 Present Address: National Synchrotron Radiation Research Center, Hsin Chu, Taiwan

**Keywords:** DNA, Resistive switching, Metallization

## Abstract

DNA-templated metallization is broadly investigated in the fabrication of metallic structures by virtue of the unique DNA-metal ion interaction. However, current DNA-templated synthesis is primarily carried out based on pure DNA in an aqueous solution. In this study, we present in situ synthesis of metallic structures in a natural DNA complex bulk film by UV light irradiation, where the growth of silver particles is resolved by in situ time-resolved small-angle X-ray scattering and dielectric spectroscopy. Our studies provide physical insights into the kinetics and mechanisms of natural DNA metallization, in correlation with the multi-stage switching operations in the bulk phase, paving the way towards the development of versatile biomaterial composites with tunable physical properties for optical storage, plasmonics, and catalytic applications.

## Introduction

The demands for sustainable technology solutions based on renewable materials has grown substantially in recent years. In that aspect, DNA as natural biopolymer, which can be readily extracted from variety of natural sources, has attracted considerable attention. Although natural DNA applications vary from drug delivery and biosensor design [1], those are mainly limited to short viral DNA or chemically produced DNA and require extensive purification. Other types of natural DNA, such as salmon sperm extract, have been successfully processed into thin-film devices useful for photonics and electronics [2]. The growing interest of developing natural DNA as an optoelectronic material is owing to the fact that DNA possesses several distinguished properties that are not readily achievable by other natural materials. It contains several chemical groups that can interact with various molecular and ionic structures through hydrogen bonding, coordinative forces and $\pi$-$\pi$ stacking, and in addition the sugar-phosphate backbone of DNA is negatively charged, aiding the electrostatic interaction with various cationic compounds [3]. The resulting DNA complexes have been shown to exhibit tunable optical, mechanical, and electric properties [4–6]. In addition, unlike natural DNA, they can be processed using common organic solvents and exhibit more robust physiochemical properties under a range of environmental conditions. Easy of processing rendered natural DNA highly compatible with several deposition platforms such as spin coating or casting. Structurally, the unique helical structure of double stranded DNA can serve as an excellent scaffold, for example, for organization of functional chromophores. This feature is widely exploited in developing efficient light-emitting devices [7], laser gain media [8, 9], and resonance energy transfer (FRET)-based applications [10, 11]. Another attractive feature of DNA is its interaction with a range of metal cations, which lead to advances of DNA-mediated synthesis and nanofabrication. DNA-templated metallization in solution has been used to prepare elements such as conductive wires and nanocomposite materials employing either natural or sequence specific small DNA sequences. Although in-solution DNA mediated structures have been successfully prepared, they are generally difficult to translate into fabrication of solid bulk photonic and electronic devices. As a results, it is highly desirable to develop and understand strategies in which natural DNA is processed to form bulk films by facile deposition methods, and then used for growth of metallized structures. Interestingly, despite extensive exploration of DNA metallization in solution, limited studies have been performed to explore this process within a bulk film. Previously, we demonstrated that, once deposited on electrode, DNA film can further be used to grow silver and gold nanoparticles using phototriggered strategies [12, 13]. However, in order to further advance the field of DNA-based devices and tune the properties, it is crucial to understand the kinetics and underlying mechanism of DNA metallization in bulk.

**Fig. 1:**
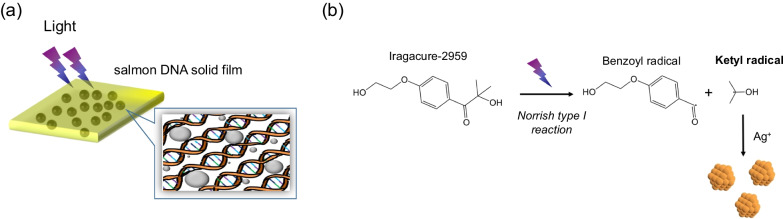
**a** Illustration of phototriggered DNA metallization in a solid matrix **b** Under UVA light, the photoinitiator I-2959 produces free ketyl radicals via Norrish-type-I $\alpha$-cleavage. The ketyl radicals function as reducing agents for the reduction of silver ions

In this study we present an in-depth investigation of UV-light driven DNA metallization process in a solid state, which is based on in situ photoreduction of silver ions in a solid DNA film (Fig. 1a). The analysis of the metallization process by synchrotron X-ray scattering and time-resolved impedance enabled us to elucidate the kinetics and mechanism of particle evolution within a natural DNA matrix. In addition, detailed optical and morphological characterization of DNA composite is provided giving more insight into the kinetics and mechanism of metallization process. Presented data not only enhances the fundamental understanding of DNA bulk film behavior but might lead to more rational design of biomaterial based devices for implantation in electronics and photonics.

## Methods

### Materials

Deoxyribonucleic acid (DNA) from salmon testes (D1626), silver trifluoroacetate (CF$_3$COO-Ag) and photoinitiator Irgacure-2959 were purchased from Sigma-Aldrich. The surfactant, cetyltriqmethylammonium chloride (CTMA), was purchased from Alfa Aesar. DNA and CTMA were dissolved in deionized water with a concentration of 3.9 g/L and 7.8g/L, respectively, followed by stirring for one day at room temperature. The molecular weight of DNA was adjusted by sonication to be 1500 kDa using a probe sonicator (Q700, Qsonica). The DNA solution was added to the CTMA surfactant solution at a ratio of 1:2 (w/w) and the mixed solution was stirred at 50 $^\circ$C for 6 hours. The solution was filtered by the centrifuge (Z206A, Hermle) for 30 minutes at 6000 rpm and the precipitates of DNA-CTMA complex were collected. The procedures of rinse and centrifuge were repeated several times. Then, the precipitates of DNA-CTMA were dried to a powder form in an oven at 40 $^\circ$C.

### Sample Preparation and Photo-Reduction Process

The DNA-CTMA composite was prepared by mixing the solution of photo-initiator I-2959(101.2g/L) and metal salt CF$_3$COOAg(33.267g/L) with DNA-CTMA(78g/L) at a ratio of 1:3:1(v), where ethanol was used as the solvent. The as-prepared solution was casted on a quartz substrate (1 square centimeter) and the casted film was dried at room temperature for 6 hours. The photo-reduction of silver nanoparticles was performed based on a photochemical synthesis [14, 15]. Under irradiation of UVA light, the photoinitiator I-2959, commonly used in polymer chemistry, produces free ketyl radicals via Norrish-type-I $\alpha$-cleavage as shown in Fig. 1b. The ketyl radicals then function as reducing agents, enabling the reduction of silver ions to silver atoms.

### Optical and Morphological Characterization

The absorption of the films was measured by a UV-VIS spectrometer (Lambda 35, Hitachi). The optical spectrum of the film was recorded at various time intervals of photo-irradiation using an UV-LED light source at 365 nm (NCSU276A, Nichia). The UV light was set above the sample with a distance of 10 cm. The optical intensity of the light source is 4.5 mW. TEM images of the films were obtained using an electron microscope (JEOL-2100) at an accelerating voltage of 200 kV. The composite films were first photo-irradiated for 60 minutes. After irradiation, the DNA sample was prepared by a lift-out technique using a dual-beam focused ion beam system (FEI Helios Nanolab 600i). A protective Ag layer was deposited on the film to prevent the composites from being damaged by the high energy ion beam.

### In Situ Time-Resolved SAXS Measurement

In situ time-resolved small-angle X-ray scattering (SAXS) measurements for the films were performed on Beamline 25A of the Taiwan Photon Source storage ring at the National Synchrotron Radiation Research Center (NSRRC) in Hsin-Chu, Taiwan. The sample-to-detector distance was set to be 4.078 m and the X-ray wavelength was $\lambda =$ 1.4Å. The sample was mounted in a holder with arrays of holes for X-ray penetration and for reducing the scattering noise from the substrate. During the measurement, the sample was irradiated by the UV-LED light source which was set slightly off-axis from the X-ray path. After receiving data every 30 s for 1 hour, while moving the measuring position in a grid around the sample, the 2D data were obtained by circularly averaging the 1D SAXS profiles. All measurements were carried out at room temperature.

SAXS modeling was performed using a modified Beaucage model [16–18], which consisted of a fractal with Guinier cut-off combined with a polydisperse sphere form factor. The scattering intensity is given by

1 $$\begin{aligned} I(q)=Gq^{-\alpha }\exp \left( {\frac{-q^2 R_g p^2}{3}}\right) + B\int f \left( r;{\bar{r}},\sigma \right) F^2(q,r) \mathrm {d}r + bkg. \end{aligned}$$

The first part of Eq. (1) describes a power-law with Guinier cut-off and the second part is a polydisperse sphere form factor. *G* and *B* are multiplying factors, *q* is the magnitude of the scattering vector, where $q=|\vec {q}|= \frac{4 \pi }{\lambda } \sin \left( \frac{\theta }{2} \right)$, $\lambda$ is the wavelength of incident radiation, $\theta$ the scattering angle, $R_g$ is the high-*q* Guinier cut-off, *p* is a factor controlling the strength of the cut-off, and *bkg* is a factor to account for a small influence of the DNA-CTMA matrix at high-*q*. *f* is the Shulz distribution [19, 20] and is defined by

2 $$\begin{aligned} f(r) = r^z \left( \frac{z+1}{{\bar{r}}} \right) ^{z+1} \exp \left( -r \frac{z+1}{{\bar{r}}} \right) / \Gamma (z+1), \end{aligned}$$

where *r* is particle radius, ${\bar{r}}$ the mean of the distribution, and *z* is defined by

3 $$\begin{aligned} z=\left( \frac{1}{PDI} \right) ^2 - 1, \end{aligned}$$

where *PDI* is the polydispersity index, relating to the standard deviation $\sigma$ by $PDI=\frac{\sigma }{{\bar{r}}}$ . $F^2$ is the sphere form factor [21] related to the particle radius through

4 $$\begin{aligned} F=3\frac{\sin \left( qr \right) - qr\cos \left( qr \right) }{\left( qr \right) ^3}. \end{aligned}$$

### In Situ AC Impedance Spectroscopy

Dielectric spectroscopy was performed using an impedance analyzer (Solartron 1260) with a dielectric interface (Solartron 1296) for highly resistive samples. The thin-film sample with thickness of ca. 300 nm was spin-coated on the bottom electrode. The top electrode was deposited on the film. The overlapping area between the two sandwiched stacking electrodes is 9 mm$^{2}$. The measurements were done at room temperature over frequency range of 10$^{-1}$ $\sim$ 10$^{6}$ Hz. The experimental impedance spectra were fitted to an equivalent circuit model using Zview software.

## Results and Discussions

### Optical and Morphological Properties

**Fig. 2:**
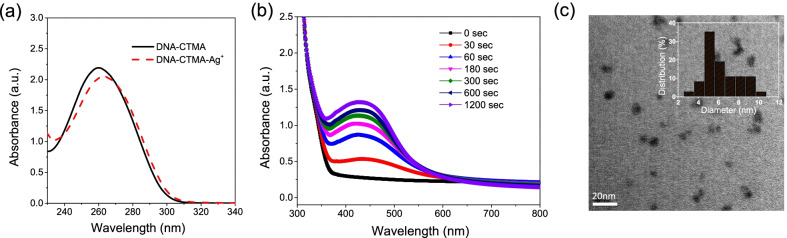
**a** Optical absorption spectra of DNA-CTMA and DNA-CTMA-Ag **b** The optical absorption of the nanocomposite films versus irradiation time for DNA-CTMA-Ag **c** TEM image of DNA-CTMA-Ag composite after the UV irradiation for 3000 s. The inset figure is the histogram of the particle radii

Silver metallized DNA was prepared using UV irradiation of silver trifluoroacetate mixed with the ethanol solution of DNA-CTMA. The absorption spectra of DNA-CTMA and DNA-CTMA mixed with the silver trifluoroacetate are shown in Fig. 2a. Before mixing, the characteristic DNA absorption peak at 260 nm can be observed. The absorption peak undergoes a slight shift due to the binding of silver ions to DNA after addition of the silver salt [22]. The samples were then irradiated by UVA light for different durations. The optical spectrum changes and an absorption band around 435 nm emerges and comes more prominent over the course of UV light irradiation (0–1200 seconds, Fig. 2b). This absorption band is attributed to the plasmonic resonance peak of the photo-reduced Ag NPs. The absorbance increases with the time of irradiation particularly during the first 3 minutes, followed by slower changes from 5 to 20 minutes. The characteristic shape of the spectral profile is maintained throughout the UV irradiation process. The TEM study of DNA-CTMA-Ag composite after UV irradiation for 3000 seconds shows that Ag NPs of roughly 4-9 nm in size (average diameter is 6.3 nm) are uniformly dispersed in the DNA matrix as shown in Fig. 2c. The inset figure is the histogram of the particle radii. This narrow size distribution of small Ag NPs achieved within the solid matrix is remarkable. Small Ag NPs are not only interesting due to their plasmonic properties but have been increasingly employed in design of remarkable catalytic systems [23], indicating that our strategy has a potential beyond the use in optoelectronic devices.

### In Situ Time-Resolved SAXS

**Fig. 3:**
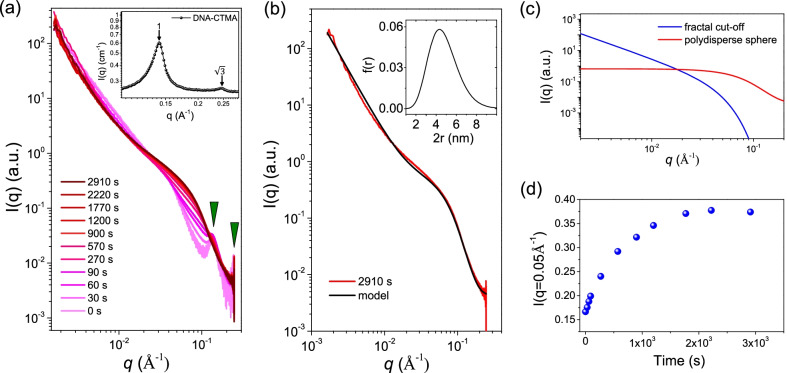
**a** Time-resolved SAXS profiles of DNA-CTMA-Ag composites under UV irradiation. **b** Model fitting of time resolved SAXS data at $t=2910$ seconds to Eq. (1). The insert shows the Shulz distribution, Eq. (2), used in the fitting. **c** The two components of the model in Eq. (1) **d** SAXS intensity at $q=0.05$ Å$^{-1}$ versus UV irradiation time for the DNA-CTMA-Ag composites

To investigate both the kinetics of Ag NP growth during reduction and the final NP morphology SAXS was employed. Due to the high electron density of the Ag NPs, their scattering length density (SLD) is significantly higher than either DNA or CTMA with values of 77.9, 15.3 and 4.87 $\times 10^{-6}$ Å$^2$, respectively. Such a difference in SLD between the inorganic and organic phase results in a strong signal from the nanoparticles embedded in the DNA-CTMA matrix. Before UV irradiation (t $=$ 0 second), the SAXS profile of the DNA-CTMA-Ag composite shows two diffraction peaks with a ratio of 1:$\sqrt{3}$, revealing that the assembly is arranged in a hexagonally packed cylinder conformation, as is shown in Fig. 3. Such hexagonal assemblies are consistent with the two diffraction peaks observed in the SAXS profile of the pristine DNA-CTMA complexes as shown in the inset of Fig. 3a. These results indicate that the cylinder-like DNA-CTMA-Ag complexes maintain hexagonally packed ordering before photo-reduction. During UV irradiation, an increase in the scattering intensities over the *q* range of $0.03 - 0.14$ Å$^{-1}$ with little changes in intensity at lower *q* was observed. This is consistent with the formation of isolated spherical particles, where the significant change in scattering is within the region corresponding to the form factor for isolated spheres [24–26]. If significant aggregation or inter-particle interaction was observed, an increase in the low-*q* scattering intensity would be seen together with a peak-like distortion at the *q* position corresponding to the particle diameter [24–26]. To both quantify the final particle size and further highlight that the scattering is indeed related to isolated particles, the measurement from 2910 seconds was fitted to Eq. (1) as shown in Fig. 3b. The model consists of two parts, a power law with Guinier cut off, which accounts for primarily DNA scattering at low-*q*, and a polydisperse sphere form factor for the Ag NPs. Figure 3c shows the fitting of the two parts of the equation to clarify the contribution from each. Values used in fitting are as follows: $G=7.5 \times 10^{-5}$, $\alpha =2.3$, $R_g=100$ Å, $P=5$, $B=3.5 ^{-6}$, ${\bar{r}} = 23.8$ Å, $PDI = 0.3$, $bkg=3.5 \times 10^{-3}$, and 100 points were used in the Schulz distribution. The model revealed that the nanoparticles have an average diameter of $2r=4.8$ nm with a size distribution described by a Schulz distribution of PDI $=$ 0.3. The low-*q* region was characterized by a power law with a slope of -2.3, indicating some larger length-scale density heterogeneity. A power-law slope of -2 corresponds to a mass fractal of 2 and can be considered a random distribution of scatterers [27, 28]. The fitting result agrees well with the size and shape extracted from TEM and furthermore gives clear insights with the absence of sample preparation, sampling bias, and image distortion which can occur in TEM micrographs [29].

During photo-reduction, SAXS further revealed a number of changes to both the DNA-CTMA matrix and the growth of nanoparticles. It can be seen in Fig. 3a that there is a complete disappearance of the first order diffraction peak in the DNA structure factor at reduction times greater than 570 seconds. This indicates that the growth of NPs significantly distorted the DNA-CTMA matrix. Changes at large length-scales can further be observed in the low-*q* fractal scattering, where an increase of the mass fractal from 2.0 ($0.0025< q < 0.01$ Å$^{-1}$) to 2.3 shows that NP growth leads to scattering, consistent with heterogeneity at large length-scales. It is not clear whether this is a result of the DNA distortion alone or the NP growth being more concentrated to regions in the sample. The high-*q* SAXS results show that the NPs are isolated, although the low-*q* results could indicate that the location of NP growth is not random but instead the NP number density fluctuates through the sample. The time resolved SAXS data can furthermore provide insights into the growth rate of the isolated NPs as the intensity is related to the NP number and size through $I(q) \propto NV^2$, where N is the number of nanoparticles in the scattering volume and V is the NP volume. Subsequently, by monitoring the SAXS intensity at a *q* value (e.g., $q=0.05$ Å$^{-1}$) with time, the temporal increase of NP diameter can be highlighted as shown in Fig. 3d. To directly obtain the change in nanoparticle size, one must remove the effects of the DNA-CTMA matrix, which was not only the dominant scatterer at shorter reduction times, but also changed during the reduction making such a subtraction not possible.

### Real-Time Dielectric Relaxation and Conductivity

**Fig. 4:**
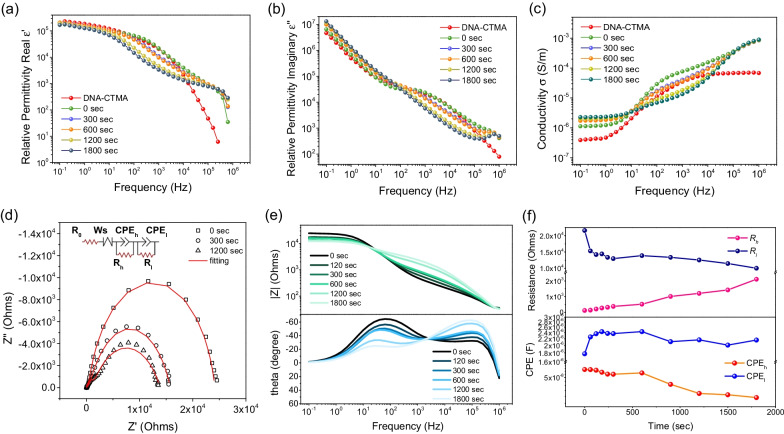
The real part (**a**) and imaginary part (**b**) of the dielectric permittivity for the pristine DNA-CTMA and DNA-CTMA-Ag with varied UV irradiation times **c** Conductivities of the DNA-CTMA-Ag with varied UV irradiation times **d** The Cole-Cole plot of DNA-CTMA-Ag with varied UV irradiation times **e** The Bode plot of DNA-CTMA-Ag (**f**) Fitting parameters of the circuit model in the inset of **d**

To improve the understanding of Ag NP growth on DNA chain dynamics and dielectric properties, the AC impedance measurements under UV irradiation were carried out. The complex dielectric permittivity can be expressed as $\varepsilon ^{*}$=$\varepsilon ^{\prime }$+j$\varepsilon ^{\prime \prime }$, where the real part $\varepsilon ^{\prime }$ and imaginary part $\varepsilon ^{\prime \prime }$ represent the dielectric storage and loss, respectively. Figure 4a and b show $\varepsilon ^{\prime }$ and $\varepsilon ^{\prime \prime }$ for the DNA system irradiated from 0 to 1800 seconds in comparison with the pristine DNA-CTMA. As shown in Fig. 4a, $\varepsilon ^{\prime }$ decreases with frequency and the slope of each curve declines in a section-wise manner, where $\varepsilon ^{\prime }$ initially decreases slowly, followed by a few more abrupt decline sections. Figure 4b shows the $\varepsilon ^{\prime \prime }$ spectra, which display a steady decrease before 10$^2$ Hz, followed by a plateau and additional decrease. Such spectral profiles are governed by the dielectric relaxation of the materials. The relaxation behaviors can be quantitatively evaluated by the relaxation time ${\tau }$ as 1/($2\pi f_{m}$), where $f_{m}$ is the maximum frequency of the $\varepsilon ^{\prime \prime }$ peak. For the pristine DNA-CTMA, the dielectric relaxation is associated with the DNA backbone, which interacts electrostatically with CTMA as side chains via ionic interactions [30–33]. The $\varepsilon ^{\prime \prime }$ peak is evaluated in the plateau region around 10$^3$ Hz, corresponding to a relaxation time of 159 $\mu$s. With addition of Ag ions into DNA-CTMA complexes, the $\varepsilon ^{\prime \prime }$ peak at 0 second observed at ca. 2000 Hz (corresponding to ${\tau }$= 80 $\mu$s), indicating that the Ag ions interact with DNA base pairs and largely accelerate DNA chain motions [34]. Subsequently, the $\varepsilon ^{\prime \prime }$ peak slightly shifts to low frequency (from 2000 Hz to a few hundreds Hz) with increased irradiation time. This phenomenon implies that growth of Ag NPs induces a jamming effect to further suppress the dipolar motion of DNA-CTMA complexes, resulting in the relative stretched or confined conformation of DNA with a longer relaxation time (${\tau }$) in the range of 160$\sim$1600 $\mu$s. Similar observation has been reported by Pungetmongkol et al. [35]. The slower motion of movable dipoles or segmental relaxation of DNA-CTMA complexes also indicate slowing down of the Ag NPs growth rate as shown in Fig. 3c. Furthermore, the Ag NPs or excess of Ag ions may bring build-ups of ionic charges on electrodes or Ag NP surface, giving rise to interface polarization (e.g., Maxwell Wagner - Sillars effect) [36–40] at low frequencies (<1 Hz). Figure 4c shows the variation of AC conductivity $\sigma$ as a function of frequency for the DNA-CTMA. The value of $\sigma$ is calculated from $\sigma$ = $\omega \varepsilon _{0} \varepsilon ^{\prime \prime }$, where $\omega$ is the angular frequency. The conductivity spectra at lower frequencies (below ca. 1 Hz) possess a frequency-independent plateau, followed by a two-step-like power law behavior ($\sigma$ $\sim$ $f^{s}$) at high frequencies (above 10 Hz). The power-law behavior changes with the irradiation time, indicating that the conductive properties of DNA are affected by the growth of Ag NPs [41–43].

The corresponding Cole-Cole plots are shown in Fig. 4d. There are two semicircles, where the larger one is at low frequencies and the smaller one is at high frequencies. Such two-mode behavior can also be clearly observed in the evolution of phase angle in the Bode plot in Fig. 4e. The phase peak at low frequencies (several tens of Hz) gradually decreases with UV irradiation, whereas the other peak at higher frequencies (ca. $10^{5}$ Hz) increases. The Cole-Cole plot was fitted by an equivalent circuit as depicted in the inset of Fig. 4d. The circuit model consists of one Ohmic resistance of electrode ($R_{o}$) in series with one Warburg impedance (Ws) along with two sets of R-CPE circuits in parallel, where $R_{h}$ and $R_{l}$ are the two charge transfer resistors, and CPE$_{l}$ and CPE$_{h}$ are the constant phase elements. The subscripts *l* and *h* represent the parameters obtained from the large semicircle at low frequencies and small semicircle at high frequencies, respectively. The fitted values of $R_{h}$ and $R_{l}$ with respect to irradiation time are shown in Fig. 4f. The signal at the low frequency is relevant for the conductivity of the composite, which is directly correlated to the growth of Ag NPs, whereas the signal at the high frequency is associated with the segmental relaxation behaviors of DNA-CTMA [44, 45], which is affected by the presence of surrounding Ag NP and Ag ions [46, 47]. As the irradiation time increases, the concentration of Ag ions gradually decreases as they are used up for the formation of Ag NPs. From the parameters at the low frequency, we can see $R_{l}$ gradually decreases with irradiation time, whereas the capacitance CPE$_{l}$ increases. This implies the growth of Ag NPs and accumulation of Ag ions at the NP boundary, in line with the SAXS analysis. However, the growth of Ag NPs further leads to distortion of the base packing of DNA chains and, as a result, the resistance $R_{h}$ gradually increases with irradiation time.

### Phototriggered DNA Metallization and Resistive Switching Effects in the Bulk Phase

**Fig. 5:**
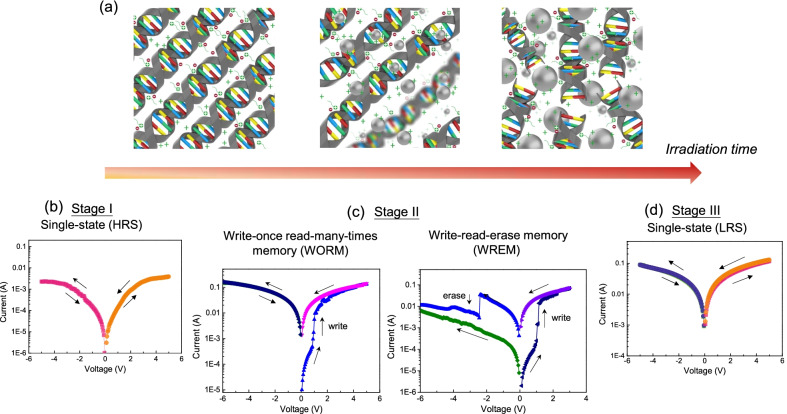
**a** Illustration of the phototriggered metallization process in the solid DNA-CTMA-Ag matrix. Depending on the irradiation time, the DNA devices exhibit different resistive switching scenarios with single-state in **b** and **d**, and WORM-type/WREM-type operations in **c**

Based on the results of characterizations, kinetic growth behaviors of Ag NPs during the photo-reduction process are illustrated in Fig. 5a. In the DNA-CTMA-Ag composite, the silver precursor and I-2959 are uniformly distributed in the hexagonally-packed DNA-CTMA mixture. In the beginning of the UV irradiation process, reduction of the silver precursor results in formation of Ag nuclei. With continuous UV irradiation, the rapid photo-reduction leads to an increase of Ag NP nuclei along with induced abundant nuclei distributed in the DNA-CTMA matrix. In the process, some Ag ions position themselves within the double helical DNA chains to form ion channels. Such positioning may facilitate the formation of conductive channels between Ag NPs and electrodes, resulting in the change of the electrical properties. In a later stage of photo-reduction, the precursor and the reducing agent are largely depleted in the matrix. In this stage, there is no considerable change in the particle size and nucleation density of Ag NPs.

It is informative to associate the evolution of the composite with the corresponding electrical properties during the metallization process. Previously we carried out electrical characterization of the phototriggered DNA composites based on a simple sandwiched structure, consisting of the composite sandwiched by two electrodes [48]. We did observe multi-stage resistive switching scenarios depending on the light irradiation time, revealing four types of electrical characteristics as illustrated in the lower panel of Fig. 5. With short light irradiation in Fig. 5b or prolonged light irradiation in Fig. 5d, the device exhibits only one single resistance state, that is, the high-resistance state (Stage I: HRS) and the low-resistance state (Stage III: LRS), respectively. The HRS in the initial stage reveals the insulating nature of the DNA-CTMA dielectric layer with a low degree of photo-reduction. For prolonged irradiation, the Ag NPs are formed and dispersed in the DNA-CTMA matrix, where conduction channels are readily available upon applied voltages and the device exhibits a low resistance character. Between the two single-state operation scenarios, there is an intermediate phase where the devices may exhibit resistive switching responses with write-once read-many-times memory (WORM)-type or write-read-erase memory (WREM)-type behavior (Stage II) in Fig. 5c. The former operation represents the case where the devices can only be switched from HRS to LRS once and never return to the HRS by subsequent sweeps of either polarity. It is attributed to the construction of non-reversible conduction paths in the dielectric layer. The latter operation represents the case where the devices can be switched between the HRS and LRS repeatedly. It is ascribed to a reversible process of formation/rupture of the conducting filaments in the dielectric layer. As described in the metallization process, abundant Ag NP nuclei and ions may facilitate the formation of the conducting filaments in this stage, resulting in WORM- or WREM-type operation. From the demonstration, we can see one of the attractive features of the in situ synthesis is that the composite properties can be further tailored by a post-fabrication illumination procedure, providing an additional degree of freedom to manipulate the optoelectronic properties after device fabrication.

Finally, to further justify the role of DNA, we carried out the same in situ phototriggered experiment based on poly(methyl methacrylate)(PMMA), which is a common polymeric material for resistive switching devices [49]. As a comparative study, the preparation and characterization follow the same procedures as previously described for DNA-CTMA-Ag. The experimental details are in the supplemental materials and the characterization results are shown in Additional file 1: Fig. S1. The absorption spectra of the PMMA-Ag film reveal that several absorption peaks emerge during the irradiation process, indicating various plasmonic resonances ascribed to irregular formation of Ag NPs. This is in line with the TEM image of the PMMA-Ag composite, which shows multiple Ag NPs are grouped as clusters of various sizes in the PMMA matrix. Such nonuniform distribution of Ag NPs largely affects the electrical properties of the composite under applied bias. We observed that the electrical properties of the PMMA-based devices mostly exhibit either insulting or conducting behavior under photo-irradiation. It indicates that the conducting paths are formed incompletely between electrodes or directly tunnel through the dielectric under bias. As the formation of conducting filaments results from ionic migration driven by the electric field across the particles, irregular Ag NP sizes and nonuniform distribution of clusters may lead to more extreme potential gradients across particles, hindering the construction of reversible conducting paths in the dielectric matrix [50, 51]. In addition, we also examined the phototriggered responses using only the surfactant (CTMA) as the matrix. With only CTMA, the film is less robust with poor film quality. The absorption spectra of the film also do not show pronounced peaks under photo-irradiation (Additional file 1: Fig. S2), which further elucidates the critical role of DNA in the seed-mediated growth of Ag NPs. Our approach of DNA metallization in the bulk phase provides new strategies for DNA-based memristive technology, which also paves the way for versatile and functional biocomposites in a broad range of applications.

## Conclusions

In this study, we presented in situ characterization of the DNA metallization process by photo-reduction in the bulk phase. Our study provides critical insights toward a well-controlled photo-reduction composite system implemented in natural DNA biopolymer. The morphological features of the composite, stemming from the templating characteristic of the DNA matrix, may be highly associated with the versatile operation scenarios in DNA-based memristors. The DNA metallization may also be of great interest for more advanced development based on natural DNA biopolymer and composites for optical storage, plasmonics, and catalytic applications.

## Supplementary Information

**Additional file 1: **In situ phototriggered experimental characterizations based on poly (methyl methacrylate) (PMMA).

## Data Availability

All data generated or analyzed of this study are included in this article and the Additional file 1.

## Abbreviations

DNA: Deoxyribonucleic acid
TEM: Transmission electron microscope
SAXS: Small-angle X-ray scattering
WORM: Write-once read-many-times memory
WREM: Write-read-erase memory
HRS: High resistance state
LRS: Low resistance state
NP: Nanoparticle
PMMA: Poly(methyl methacrylate)
