# Supplementary material for: Kinetics and Mechanism of In Situ Metallization of Bulk DNA Films
Source: Nanoscale Res Lett. 2022 Jan 24;17:18. doi: 10.1186/s11671-022-03658-8 (PMC8787019; doi:10.1186/s11671-022-03658-8)
Supplement: Supplementary file 1 — Additional file 1: In situ phototriggered experimental characterizations based on poly (methyl methacrylate) (PMMA). [file 11671_2022_3658_MOESM1_ESM.pdf]

Supporting Information

In situ phototriggered metallization in DNA bulk film for multi-stage resistive switching devices

Zi-Hao Shi[1], Feng-Ming Hsu[1], Bradley W. Mansel[2], Hsin-Lung Chen[2], Ljiljana Fruk[3], Wei-Tsung Chuang[4], and Yu-Chueh Hung[1,]*

[1]Institute of Photonics Technologies, National Tsing Hua University, Taiwan
[2]Department of Chemical Engineering, National Tsing Hua University, Taiwan
[3]Department of Chemical Engineering and Biotechnology, University of Cambridge, Cambridge, UK
[4]National Synchrotron Radiation Research Center (NSRRC), Hsinchu, Taiwan

Email: ychung@ee.nthu.edu.tw

1. Phototriggered Experiment of PMMA-based System

Material preparation: Polymethyl methacrylate (PMMA) (MW=15000) was purchased from ACROS Organics. The composite was prepared by mixing the solution of photo-initiator I-2959 (101.2g/L) and metal salt CF_3COOAg (33.267g/L) with PMMA (78g/L) at a ratio of 1:3:1(v) in acetone. The film deposition and photo-irradiation procedures are the same as the DNA-CTMA-based samples.

Fig. S1(a) is the absorption spectra of the PMMA system. With 0.5 min of irradiation, an absorption band is displayed around 432 nm. As the irradiation time increases, multiple absorption peaks emerge at a shorter wavelength around 377 nm and longer wavelengths around 456 nm and 498 nm, which are ascribed to the excitation of extra modes in NP aggregates. The peaks at 377 nm and 498 nm grow monotonically with irradiation time. After 20 minutes of irradiation, the absorption shows a broadened spectral profile. Fig. S1(b) is the TEM image of PMMA systems after photoirradiation for 60 minutes. We can observe multiple Ag NPs are grouped as clusters of various sizes in the PMMA matrix. One cluster region is enlarged, which displays several small Ag NPs aggregated in an area with close proximity. The histogram of particle sizes is shown in Fig. S1(c). The NP diameter ranges in a wider spectrum with a maximum diameter up to 22 nm. Multiple clusters of NPs are randomly distributed in the PMMA matrix with an average size of 75 nm.

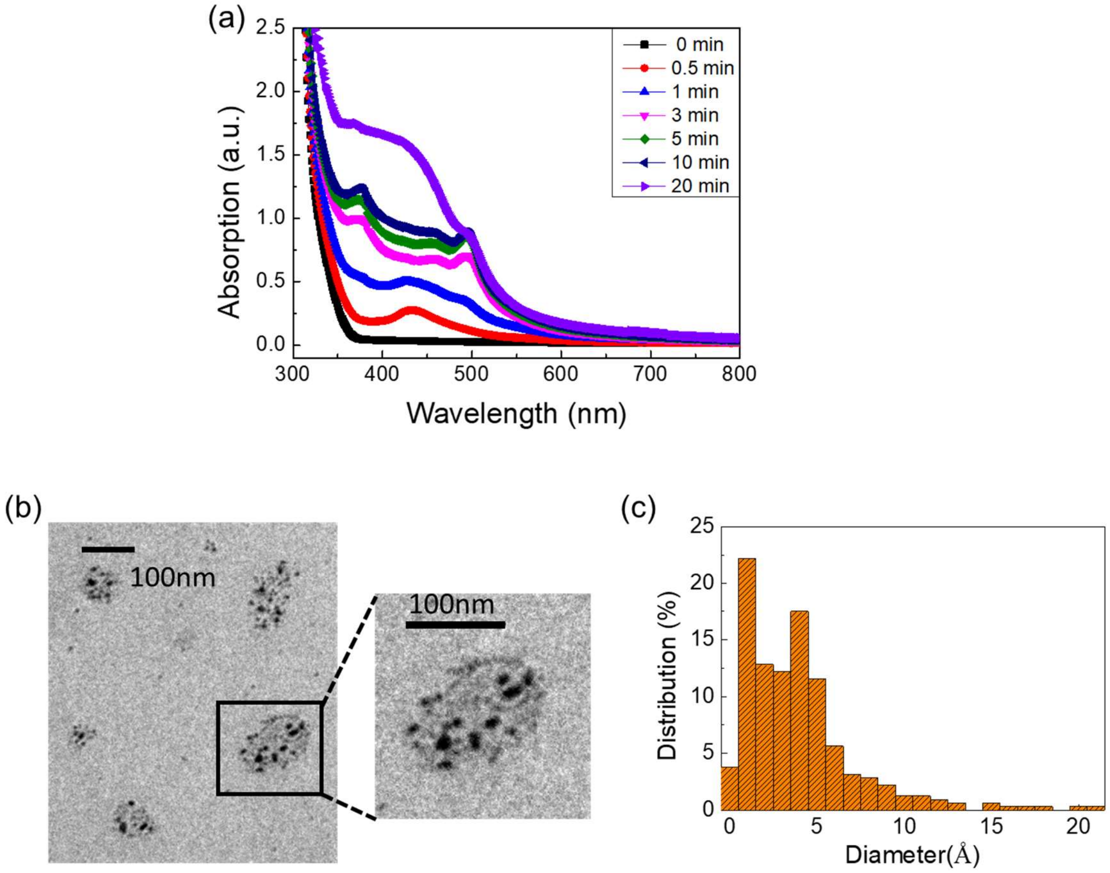

Figure S1. (a) The optical absorption of the PMMA/Ag composite under different photo-

irradiation time. (b) TEM images of the PMMA/Ag sample. One cluster region is enlarged, which displays several small Ag NPs aggregated in an area with close proximity. Multiple clusters of NPs are randomly distributed in the PMMA matrix with an average size of 75 nm. (c) The particle size statistics show the NP diameter ranges in a wider spectrum with a maximum diameter up to 22 nm.

2. Phototriggered Experiment of CTMA-based System

Material preparation: The composite was prepared by mixing the solution of photo-initiator I-2959 (101.2g/L) and metal salt CF_3COOAg (33.267g/L) with CTMA (291.822g/L) at a ratio of 1:3:0.6(v) in ethanol. The film deposition procedures and photo-irradiation procedures are the same as the DNA-CTMA-based samples. Due to much thicker film in one deposition, more layers were deposited to increase the film thickness.

Fig. S2 is the absorption spectra of the CTMA-based system. Under photoirradiation, no evident absorption peaks were observed after light irradiation of 30 minutes.

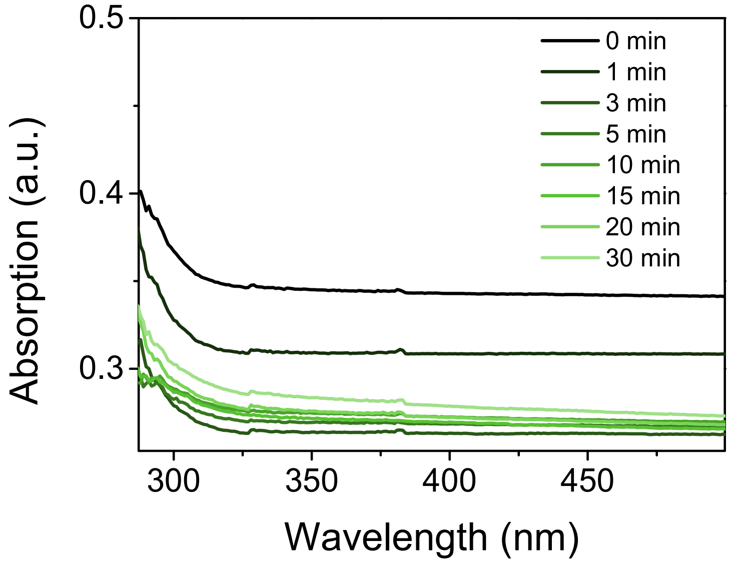

Figure S2. Absorption spectra of CTMA-based film under different irradiation time
